# Editorial: Innovations in cognitive and psychological assessment: integrating immersive VR technologies for enhanced ecological validity

**DOI:** 10.3389/fpsyg.2026.1838586

**Published:** 2026-06-03

**Authors:** Valentina Mancuso, Sara Freedman, Giovanna Calogiuri, Jorge Oliveira

**Affiliations:** 1 Department of Theoretical and Applied Sciences, eCampus University, Novedrate, Italy; 2 School of Social Work, Bar Ilan University, Ramat Gan, Israel; 3 Centre for Health and Technology, Department of Nursing and Health Sciences, University of South-Eastern Norway, Drammen, Norway; 4 HEI-Lab Digital Human-Environment and Interaction Lab, University Lusófona, Lisbon, Portugal

**Keywords:** cognitive assessment, ecological validity, psychological assessment, psychometric validation, virtual reality

The integration of immersive virtual reality (VR) into cognitive and psychological assessment represents an important methodological advance in behavioral science. Traditional tools such as paper-and-pencil tests, laboratory paradigms, and self-report measures have generated valuable insights, but they often struggle to capture cognition and behavior as they unfold in complex, dynamic, and socially meaningful real-world contexts. Immersive VR addresses this limitation by enabling realistic, interactive environments while preserving experimental control. Yet, the key question for the field is no longer whether VR feels more lifelike than traditional assessment, but whether that realism translates into measurement validity—that is, into scores that reliably reflect real-world functioning. This distinction between surface realism and psychometric validity is central to determining whether immersive tools can be meaningfully interpreted, compared across studies, and adopted in clinical practice. The contributions collected in this *Research Topic* move the field toward answering that question, while also making clear how much conceptual and methodological work remains.

This *Research Topic*, Innovations in Cognitive and Psychological Assessment: Integrating Immersive VR Technologies for Enhanced Ecological Validity, brings together six contributions spanning neuropsychological assessment, social cognition, moral reasoning, aggression, prevention, and mental health intervention. Taken together, these articles advance the field in two complementary ways. First, they move VR-based assessment closer to clinical applicability by providing normative data, formal validity evidence, and structured protocols. Second, they expose unresolved tensions—between ecological validity and experimental control, between immersion and psychometric rigor, and between technological innovation and the need for standardization. Identifying these tensions is itself an editorial contribution, because it clarifies what the next phase of the field must address.

A first major theme is the growing psychometric maturity of immersive assessment. Rebón-Ortiz et al. provide normative data for the Nesplora Ice Cream test in 419 adults aged 17–80 years across nine sites in Spain, supporting a three-factor structure centered on planning, learning, and flexibility. This contribution is important not only because it offers reference values, but because it demonstrates that ecological validity and standardization are not mutually exclusive. At the same time, the absence of convergent validation against established neuropsychological batteries leaves open the question of how closely VR-based performance relates to everyday executive functioning. This pattern is instructive: in VR assessment, psychometric and normative validation often advance faster than veridicality evidence.

A second major contribution concerns social and moral cognition. Neveu et al. present the So-Moral-VR task as an immersive assessment of socio-moral reasoning in adolescents and provide evidence for both verisimilitude and veridicality, while also reporting low social desirability effects. Their study is especially important because the VR assessment literature often claims ecological validity on the basis of realism and presence alone, without testing whether task performance predicts real-world outcomes. By linking So-Moral-VR scores to prosocial behavior, Neveu et al. provide rare veridicality evidence for a VR-based social cognition measure. More broadly, their work shows what it means to treat ecological validity not as a rhetorical advantage of VR, but as a structured validation problem. This *Research Topic* is further sharpened by Pucci and Gagliardi, whose review of VR in moral dilemma research highlights both the promise and the conceptual difficulty of immersive methods. VR can enrich moral research by incorporating embodiment, action, and multimodal data, but increased immersion does not automatically produce more valid measurement. Read together, these contributions make a crucial point: the field must move beyond asking whether VR is more realistic, and instead ask under what conditions realism improves assessment.

Sappelli et al. extend this discussion to aggression assessment by reporting preliminary evidence for the construct, known-group, and concurrent validity of a VR aggression scenario, with associations with trait aggression reaching significance only in the combined sample. Their study is valuable not only for its findings, but because it illustrates a core trade-off in immersive assessment: realistic social interaction may require flexible operator control, but this can compromise strict standardization. This is not simply a methodological weakness; it is a reminder that some constructs, especially socially reactive ones, may require a measured balance between ecological fidelity and experimental control.

The *Research Topic* also shows that immersive technologies are relevant beyond assessment narrowly defined. De Simone et al. describe a prospectively registered protocol for an immersive VR and telemedicine-based intervention for subjective cognitive decline, designed for delivery in participants' home environments; results are not yet available. This is methodologically important because it links intervention setting to daily living context and may help address the ceiling effects often observed in standard neuropsychological testing in this population. Wang et al., in turn, report significant reductions in depression, anxiety, and stress in men with social anxiety disorder following VR-based extreme sports exergaming. Although this study is more intervention-oriented than assessment-focused, it underscores a broader point relevant to this *Research Topic*: immersive environments can generate engagement and emotional salience that conventional formats often struggle to achieve. At the same time, the absence of a non-extreme VR comparison group means that the specific contribution of immersion cannot yet be isolated from exercise, arousal, or novelty effects.

Taken together, these contributions suggest three priorities for future research. First, the field must treat veridicality as a primary validation target rather than an afterthought. Presence, realism, and low cybersickness are important, but they do not by themselves demonstrate valid measurement. Second, immersive assessment requires stronger shared standards—not only for hardware and software, but also for scenario design, operator behavior, scoring procedures, and reporting practices—if findings are to accumulate across laboratories and clinical settings. Third, VR's distinctive strength lies in its ability to combine behavioral, kinematic, gaze-based, and physiological data streams within the same assessment environment. The next challenge is therefore not simply to collect multimodal data, but to develop psychometric models capable of turning those data into interpretable and clinically useful scores.

The contributions collected here mark a meaningful transition in the VR assessment literature: from showing that immersive tools can elicit psychologically relevant behavior, to asking whether and under what conditions that behavior constitutes valid measurement. Whether immersive VR ultimately becomes a standard component of psychological and neuropsychological assessment will depend not on further demonstrations of its technological capabilities, but on its capacity to meet the same standards of validity, reliability, and clinical utility expected of any assessment tool.

